# ARSOD-YOLO: Enhancing Small Target Detection for Remote Sensing Images

**DOI:** 10.3390/s24237472

**Published:** 2024-11-23

**Authors:** Yijuan Qiu, Xiangyue Zheng, Xuying Hao, Gang Zhang, Tao Lei, Ping Jiang

**Affiliations:** 1 National Laboratory on Adaptive Optics, Chengdu 610209, China; 2 University of Chinese Academy of Sciences, Beijing 101408, China; 3 Institute of Optics and Electronics Chinese Academy of Sciences, Chengdu 610209, China

**Keywords:** object detection, remote sensing, small object, feature fusion

## Abstract

Remote sensing images play a vital role in domains including environmental monitoring, agriculture, and autonomous driving. However, the detection of targets in remote sensing images remains a challenging task. This study introduces innovative methods to enhance feature extraction, feature fusion, and model optimization. The Adaptive Selective Feature Enhancement Module (AFEM) dynamically adjusts feature weights using GhostModule and sigmoid functions, thereby enhancing the accuracy of small target detection. Moreover, the Adaptive Multi-scale Convolution Kernel Feature Fusion Module (AKSFFM) enhances feature fusion through multi-scale convolution operations and attention weight learning mechanisms. Moreover, our proposed ARSOD-YOLO optimized the network architecture, component modules, and loss functions based on YOLOv8, enhancing outstanding small target detection capabilities while preserving model efficiency. We conducted experiments on the VEDAI and AI-TOD datasets, showcasing the excellent performance of ARSOD-YOLO. Our algorithm achieved an mAP50 of 74.3% on the VEDAI dataset, surpassing the YOLOv8 baseline by 3.1%. Similarly, on the AI-TOD dataset, the mAP50 reached 47.8%, exceeding the baseline network by 6.1%.

## 1. Introduction

With the rapid development of remote sensing technology, an increasing number of high-quality remote sensing images are being generated and applied to various fields such as disaster assessment, military surveillance, and automatic driving [1,2]. These images provide substantial data support for remote sensing image interpretation experts. However, the conventional methods that heavily depended on manual annotation are evidently inadequate to handle the vast amount of data present in modern images, necessitating the development of efficient algorithms for processing. Remote sensing object detection is a critical method of interpreting images, playing a central role in both the military and civilian sectors.

**Figure 1:**
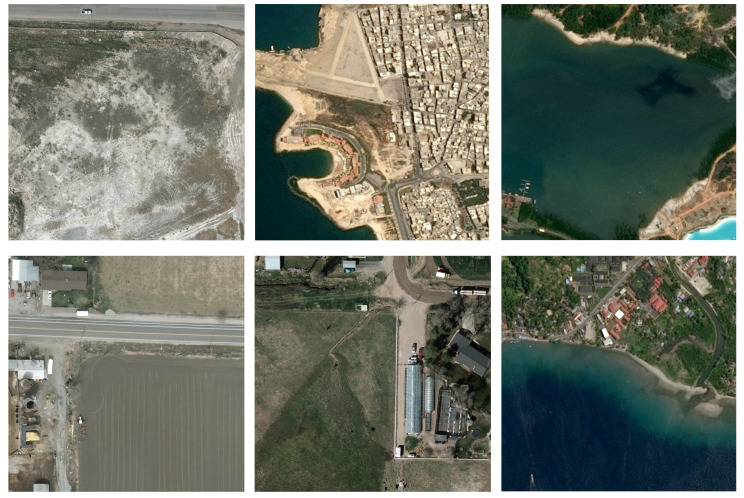
Some examples of remote sensing images.

As depicted in Figure 1, remote sensing images are different from natural images in shooting distance and image scale, presenting significant challenges [3]. However, due to the restricted size of small targets, mainstream detectors exhibit high false detection and missed detection rates [4,5].

Traditional methods for remote sensing target detection rely on manual feature design, leading to limited generalization and computationally intensive procedures, rendering them inadequate for handling vast image datasets [6,7]. With the advancement of deep learning technology, remote sensing object detection algorithms based on neural networks have gradually become mainstream. These algorithms not only avoid the shortcomings of manually designed traditional algorithms but also excel in feature extraction from extensive datasets, exhibiting high generalization. Significant improvements in both detection speed and accuracy have been realized. The popular remote sensing target detection algorithms based on deep learning can be categorized into single-stage detection and two-stage detection. The two-stage detection algorithm initially filters possible regions of the target, followed by conducting classification and regression on the proposed regions. Conversely, the single-stage detection algorithm eliminates first stage and directly conducts the detection. Therefore, the single-stage detection is generally faster, albeit at a slight cost to accuracy. The YOLO series is well-known for its speed and accuracy balance, making it a popular choice in both academic research and industry. n this study, YOLOv8 was chosen as the detection benchmark to achieve an optimal trade-off between detection speed and accuracy.

For algorithms utilizing deep learning to detect remote sensing small targets, the existing challenges can be categorized into the following three main points: (1) Extracting effective features and distinguishing small targets from the background is challenging due to insufficient features caused by long shooting distances and small sizes [8,9]. (2) Small targets often lose information during continuous convolution, emphasizing the significance of feature fusion techniques. Many existing methods manually specify convolution kernel sizes for feature detection and fusion, hindering the network’s ability to autonomously determine the most crucial kernel sizes for accurate detection. (3) The detection of small targets is more prone to positional deviations, leading to lower positioning accuracy. Additionally, remote sensing images show significant scale differences, posing a challenge for mainstream remote sensing detection algorithms that are not optimized for effectively detecting small targets.

To address these challenges, we have made improvements in the following aspects:

(1) Adaptive Selective Feature Enhancement Module (AFEM): For efficient small target detection in remote sensing images, we designed an adaptive selective feature enhancement module (AFEM) to enhance the importance and detection accuracy of small target features by dynamically adjusting feature weights and using GhostModule and sigmoid activation functions. (2) Adaptive Multi-scale Convolution Kernel Feature Fusion Module (AKSFFM): Introduced to enhance feature fusion in remote sensing object detection, the AKSFFM improves feature dependency through convolution operations at various scales and attention weight learning, enhancing the model’s ability to fuse features effectively in complex remote sensing images. (3) ARSOD-YOLO model: A model based on YOLOv8, the ARSOD-YOLO model is optimized in terms of network architecture, component modules, and loss functions. It outperforms some SOD models, offering superior detection capabilities for small targets in remote sensing while remaining lightweight.

## 2. Related Works

### 2.1. Small Target Detection Algorithm Based on Feature Enhancement

The challenge in detecting small objects predominantly arises from the possible loss of small object features due to the downsampling operation. The abstraction in high-level feature mAPs results in insufficient spatial position information for small targets. Current research addressing the insufficient feature extraction for small targets can be categorized into two approaches.

One prevalent approach involves incorporating pre-processing techniques. Super resolution technology is employed to enhance image resolution prior to detection. Zhang et al. introduced Deconv-RCNN [10], utilizing deconvolution operations on extracted feature mAPs to recover information lost during pooling layers, thereby enhancing detection accuracy. Currently, the mainstream trend involves leveraging Generative Adversarial Networks (GANs) [11] for super resolution. Courtrai [12] integrated a GAN-based super-resolution network branch to enhance input image resolution before detection. MT-GAN [13] employs a multi-task network to address target classification and detection simultaneously. Li [13] devised the Perceptual GAN to exploit intrinsic correlations among objects of varying scales. Wu proposed a point-to-region micro-target detection framework [14] that predicts candidate regions using keypoints and applies a multi-task GAN for super-resolution processing. Additionally, Ma [15] enhanced feature distinctiveness by establishing connections between small objects and their surroundings through self-attention mechanisms.

One strategy involves enhancing small target features by leveraging prior knowledge of semantic and spatial associations based on contextual information. Zhao [16] introduced the Reception Field Block (RFB) module to enhance feature expression capabilities. Luo [16] employed the CSandGLass module to replace residual modules in the Backbone network, enhancing aircraft detection performance. Bell [17] utilized jump connections and spatial recursion networks to capture crucial information inside and outside the region of interest, thereby enhancing target detection accuracy. SCRDet [18] integrates two attention mechanisms to improve effective feature representation and suppress irrelevant noise. PyramidBox [19] combines high-level contextual semantic features with low-level texture features to identify small objects efficiently.

### 2.2. Small Target Detection Algorithm Based on Feature Fusion

Numerous researchers have explored synthesizing shallow and deep features. They emphasize fusing features from various network branches to incorporate both deep semantic insights and shallow detail information, culminating in enhanced feature representations for detecting small targets.

The Feature Pyramid Network (FPN) introduced by Lin [20] pioneers top-down feature fusion, linking low-level and high-level feature graphs rich in semantic content. PANet [21] has achieved increased accuracy on specific datasets by incorporating bidirectional paths for enhancement. ASFF [22], NAS-FPN [23], and RefinDet [24] have also demonstrated notable results. BiFPN [25] proposes a bidirectional weighted feature network, learning scale-specific weights for effective cross-scale fusion. Lim et al. [26] leveraged varying layers of context information and attention mechanisms to boost small target detection accuracy. Liu et al. [27] proposed two pyramid networks to extract features efficiently while reducing computational load. Nie [28] improved the YOLOv8 Neck network by merging up-sampled deep feature mAPs with shallow ones. Zhao [29], when detecting UAVs, integrated more feature layers through cross-connections, enriching semantic information. FMSSD [30] emphasizes small target detection accuracy via area weighting functions. FS-SSD [31] combines feature fusion and multi-scale scaling to effectively detect tiny UAV targets. Bai et al. [32] devised a bidirectional pyramid network for infrared small-size targets, enhancing the original FPN structure with a bottom-up pyramid network for richer feature fusion. Li et al. [33] proposed an attention network for context extraction, bolstering detection efficacy. Liu et al. [33] introduced IPG-NET and the IPG transformation module to supplement spatial information through feature extraction at varying resolutions. Gong [34] innovated a statistical fusion factor to regulate feature flow across adjacent layers.

### 2.3. Remote Sensing Small Target Detection Method Based on YOLO

The YOLO (You Only Look Once) series has found extensive applications in remote sensing target detection. Cao et al. [35] developed a detection head tailored for remote sensing small objects, enhancing a lightweight network based on GhostConv. Liu et al. [36] integrated the CBAM into the YOLOx network. Li et al. [37] combined attention mechanisms with the MobileNetv3 Backbone network to enhance YOLOV3, subsequently enhancing detection accuracy. Wan [38] introduced the YOLO-HR algorithm, employing a lightweight hybrid attention module, validated with high-resolution optical remote sensing images. Qu [39] designed a branch network to enhance features of small targets, utilizing an adaptive feature fusion technique to enhance target detection efficiency. Addressing the diverse scales in remote sensing images, Xu [40] introduced DenseNet to enhance the Backbone for improved multi-scale target detection accuracy. HRDNet [27], focusing on small target detection in UAV images, merges depth and multi-scale pyramids, cross-linking high-resolution and low-resolution features for comprehensive information extraction through shallow and deep network fusion.

## 3. Materials and Methods

### 3.1. Baseline Model

**Figure 2:**
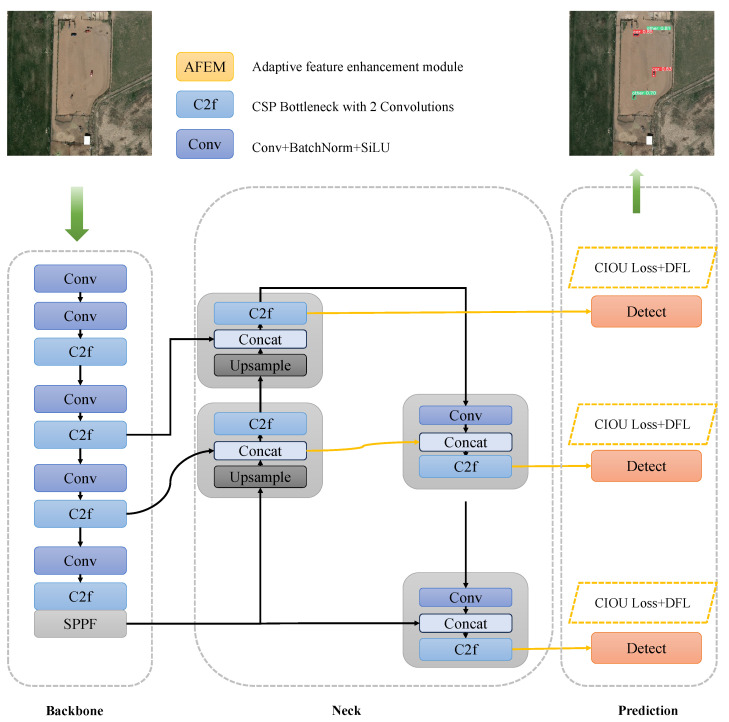
YOLOv8 network architecture.

The YOLO model is currently one of the most popular object detection algorithms. Among its iterations, the YOLOv8 model has notably surpassed previous YOLO models in all aspects. YOLOv8 has demonstrated superior detection accuracy and efficiency compared to earlier YOLO versions. The YOLOv8 network model, shown in Figure 2, can be roughly divided into three parts: the Backbone, Neck, and Prediction sections. The Backbone section is used for feature extraction, where the C2 module draws inspiration from the ELAN concept, parallelizing more gradient flow branches to obtain richer gradient flow information while ensuring lightweight design. The Neck section performs feature fusion on the features extracted by the Backbone, obtaining more context information at different scales, which has proven effective in handling tasks involving multi-scale objects. The Prediction section consists of three detection heads responsible for predicting targets. These three detection heads, respectively, detect large, medium, and small objects, providing the categories and coordinates of the targets. The YOLOv8 series is divided into five models, YOLOv8n, YOLOv8s, YOLOv8m, YOLOv8l, and YOLOv8x, each with different scales and parameters, with computational complexity gradually increasing. To achieve a good balance between speed and accuracy, in this study, we adopted the YOLOv8n model.

### 3.2. ARSOD-YOLO

**Figure 3:**
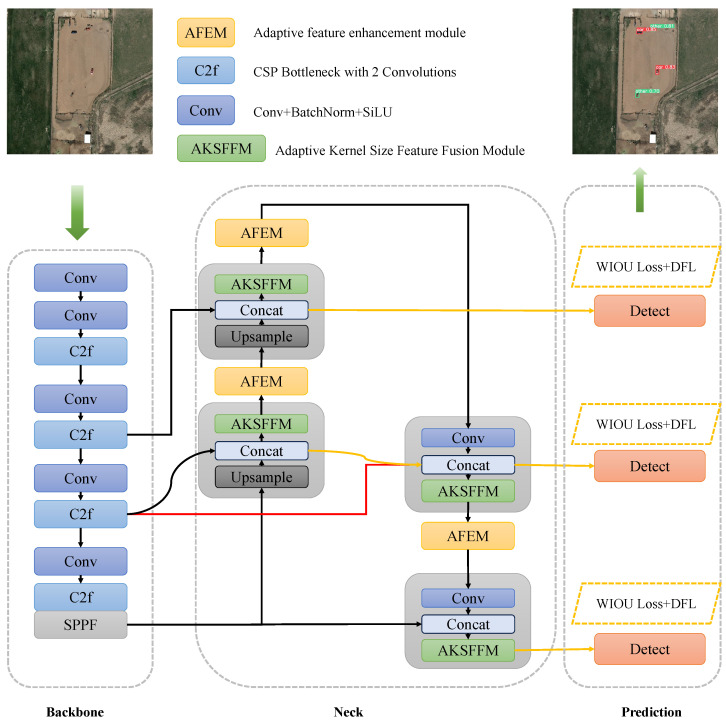
ARSOD-YOLO network architecture.

The network structure of our proposed ARSOD-YOLO (Adaptive Remote Sensing Small Object Detection) is illustrated in Figure 3. We opted for YOLOv8 as our benchmark model due to its ability to strike a balance between speed and accuracy in target detection. To enhance YOLOv8’s performance in detecting small and medium targets within remote sensing images, we implemented several improvements. Firstly, we enhanced the Neck segment by introducing the BiFPN concept and refining the original PANet network structure. Subsequently, prior to feature fusion, we integrated an Adaptive Features Enhancement Module (AFEM) at each stage to amplify small target features, represented by the yellow module in Figure 3. In the Model Feature Fusion phase, we introduced the Adaptive Kernel Size Feature Fusion Module (AKSFFM) to amalgamate context information and seamlessly integrate features across different levels, denoted by the green modules. Lastly, we employed the WIoU loss function to construct the boundary frame loss with a dynamic focusing mechanism, thereby improving the network model’s localization capability.

In refining the Neck section of the network structure depicted in Figure 3, our aim was to bolster cross-scale connections within the network architecture. Drawing inspiration from the BiFPN concept, we introduced jump connections that bridge the input and output nodes of identical scales. This strategic enhancement facilitated the incorporation of a richer array of features, as highlighted by the conspicuous red lines in Figure 3, all without imposing an undue burden on computational resources.

In the process of continuous convolution, focusing more on local information for small targets, moving the detection layers of the small and medium detection heads to the Concat layer helps to avoid potential information loss due to excessive convolution operations and enables more effective integration of features across different scales. Conversely, considering that larger targets require attention to global information, retaining the large detection head connected to the AKSFFM layer allows for the utilization of multiple convolution operations to observe large-scale features, facilitating comprehensive use of global information. Such adjustments better cater to the varying needs of targets for local and global information, thereby enhancing overall detection accuracy and performance. Specifically, we transitioned the connection layers of the small and medium detection heads to the Concat layer, thereby ensuring the preservation of crucial information. Notably, the connection layer of the large detection head remained unaltered, as indicated by the yellow line segment linking “Detect” in Figure 3.

### 3.3. Adaptive Feature Enhancement Module

Due to the nature of remote sensing images being captured from considerable distances, the targets of interest are often small within the image frame. These small targets can easily blend into the background, posing challenges for effective feature extraction. Our approach focuses on selectively amplifying features crucial for detecting these small targets. By fine-tuning the significance of features based on distinct remote sensing scenarios and target characteristics, we aim to enhance the overall capability of target detection. To achieve this, we have devised a feature enhancement module capable of dynamically choosing and enhancing features that are most relevant and effective for the task at hand.

**Figure 4:**
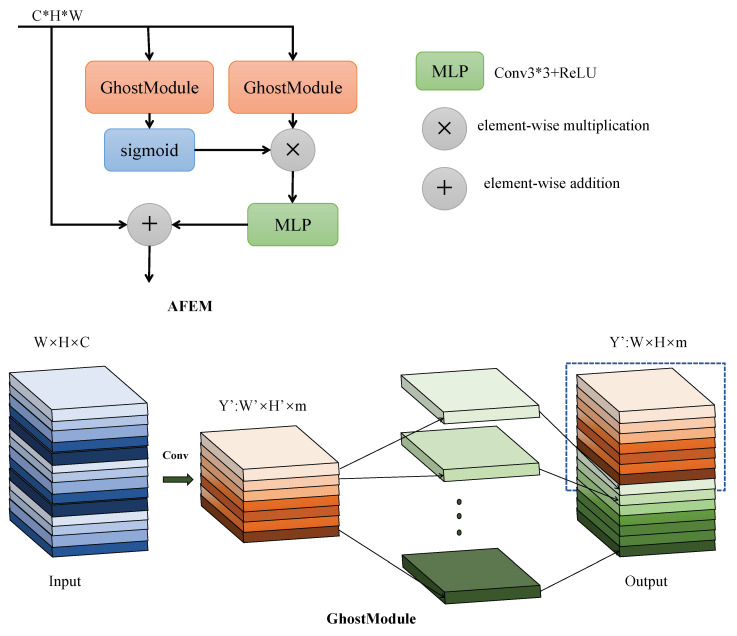
The basic structure of AFEM. It consists of GhostModule and MLP as the basic components.

In Figure 4, our Adaptive Features Enhancement Module (AFEM) initiates by establishing two branches. Each branch undergoes feature processing using GhostModule, with one branch employing a sigmoid activation function. The sigmoid function acts as a gating mechanism, facilitating the learning of correlations between diverse features and enabling the dynamic adjustment of their weights. This adaptive weighting mechanism caters to targets of varying scales and complexities. The learned importance weights in the second branch enhance the model’s understanding of target information within remote sensing images. Processing in the two branches allows the model to extract insights from different facets of the image, facilitating a comprehensive extraction of features relevant to small targets.

Subsequently, the attention-weighted features are fed into a multi-layer perceptron (MLP) to bolster the feature representation post-attention selection. This step aims to refine the remote sensing model’s understanding and detection accuracy concerning small targets. Following this enhancement, a residual operation is conducted to combine the enriched features with the initial input features, facilitating feature fusion. This process serves multiple purposes: it mitigates overfitting, preserves additional context information during feature enhancement, and equips the model with improved capabilities to address small targets within remote sensing images.

We illustrate the calculation process of the Adaptive Features Enhancement Module (AFEM) in Equation (1):

(1) $$\begin{array}{c} a=GhostModule(x,kernel\_size=1)\\ weights=\sigma \left(a\right)\\ \sigma \left(a\right)=\frac{1}{1+e^{-a}}\\ enhanced\_features=MLP(weigths\times a,kernel\_size=3,padding=1,ReLU)\\ output=x+enhanced\_features \end{array}$$

where *x* represents the input, while *a* and *b* signify the outputs after *x* undergoes GhostModule processing. σ denotes the sigmoid function used for processing. The variable σ corresponds to the weights acquired post-processing by the sigmoid functions. The term MLP refers to the multi-layer perceptron, with a kernel_size set to 3, padding set to 1, and ReLU employed as the activation function. enhanced_features signifies the resulting enhanced features.

In the initial processing of input features, it is crucial to highlight the utilization of GhostModule instead of traditional convolutions. An observation in ResNet50 revealed the presence of highly similar feature mAPs within the same residual group. GhostModule asserts that the positive correlation between highly similar features enhances CNN’s feature extraction capabilities, thereby acquiring more Ghost pairs through linear operations. The GhostModule comprises three key steps: convolution, Ghost feature mAP generation, and feature concatenation, as depicted in Figure 4. Initially, feature mAPs are derived via regular convolutions, followed by the generation of Ghost feature mAPs through linear operations (replacing linear operations with lightweight Conv). Substituting Conv with Ghost feature mAPs streamlines the process, yielding a lighter computational load. The resulting output Ghost feature mAPs are subsequently concatenated with the original feature mAPs to yield the final outcome. Compared to direct usage of regular convolutions, the GhostModule notably diminishes computational complexity while enhancing the feature mAPs derived from convolutions. The calculation formula for GhostModule is presented in Equation (2):

(2) $$\begin{array}{c} X=Conv(x)\\ Y=Conv_{linear}\left(x\right)\times W\\ Output=Concat(X,Y) \end{array}$$

where *x* represents the input, while Conv represents a common convolution, and *X* represents the features processed through the Conv. Conv_linear represents a linear operation, and Concat represents a concatenation. The weight matrix *W* is a parameter used for linear convolution operations, typically learned through the training process of the network. *Y* represents the features processed through Conv_linear with the weight matrix *W*.

### 3.4. Adaptive Multi-Convolutional Kernel Feature Fusion Module

**Figure 5:**
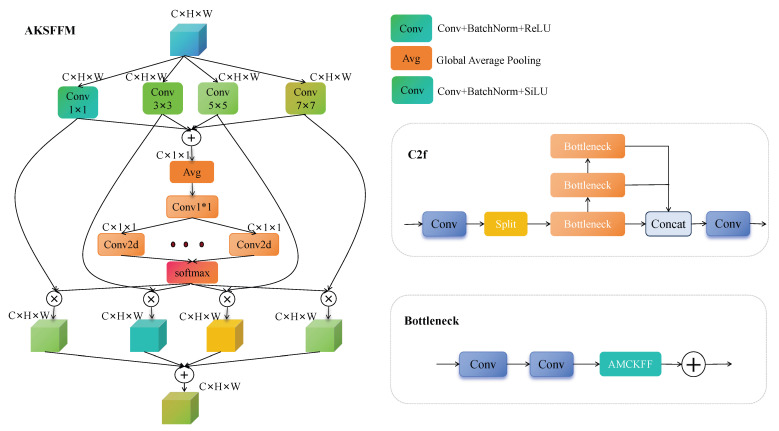
Structural diagrams of AKSFFM, C2f, and Bottleneck.

While feature enhancement has been implemented to handle small targets, remote sensing images typically exhibit a diverse array of features due to the intricate structure and textures of ground objects. It is imperative to select appropriate methods to enhance the feature fusion aspect of YOLOv8 for bolstering its fusion capacity in remote sensing target detection. The underlying features often possess higher resolution and encompass more positional information but undergo fewer convolution steps, leading to increased noise. Conversely, higher-level features, after multiple convolutions, contain richer semantic information. In response to these challenges, we introduced AKSFFM, a versatile module that dynamically selects features of varying convolution kernel sizes and processes them following each concatenation operation in the Neck section. This approach aims to effectively amalgamate feature information of diverse scales and enhance feature fusion capabilities. As depicted in Figure 5, the primary structure of AKSFFM retains the design framework of C2f but incorporates a Bottleneck for enhanced performance.

The AKSFFM module begins by employing convolution operations of 4 different scales to extract feature information of various scales from the input feature mAP, bolstering the model’s capability to perceive small targets effectively. Different kernel sizes impact the spatial range of features learned by the network and the strength of localized information. Hence, we opt for diverse kernel sizes to facilitate feature extraction and abstraction at different network levels. Subsequently, the feature mAP undergoes global pooling to derive a comprehensive global feature representation, capturing the entirety of information within the remote sensing image. To streamline computation, we utilize dimension reduction post-1x1 convolution and pooling. Attention weights for the dimensionally reduced features are computed through a series of convolutions and normalized using softmax. These weights adaptively learn the significance of each feature channel, selecting crucial features extracted from varying kernel sizes. Ultimately, by leveraging the learned attention weights, the multi-scale features obtained in the initial step are multiplied and merged with the attention weights. This process fosters interaction and information transfer across different channels, reinforcing the interdependence between features. The calculation formula for AKSFFM is provided in Equation (3):

(3) $$\begin{array}{c} Y_{k}=Conv2d\left(x,kernel=k\right),k=1,3,5,7\\ feats=Concat(Y_{1},Y_{3},Y_{5},Y_{7})\\ C=\sum_{k}Y_{k}\\ G1=Conv2d(AvgPool(C),kernel=1)\\ Weight_{k}=Conv2d\left(G1,kernel=1\right)\\ attention\_weight_{k}=Softmax\left(Weight_{k}\right)\\ Output=\sum_{k}\left(attention\_weight_{k}\times Y_{k}\right) \end{array}$$

where “*x*” symbolizes the input feature map. Here, kernel represents the size of the convolution kernel, and “$Y_{k}$” denotes the result obtained from a convolution kernel of size *k*. Furthermore, “feats” denotes the feature map acquired by concatenating features from each group, while “*C*” signifies the feature map obtained by directly summing features from each group. “*G*” represents the global feature representation derived after global pooling. “$Weight_{k}$” signifies the attention weights post-dimension reduction, whereas “$attention_{w}eight_{k}$” represents the normalized attention weights. Finally, “Output” represents the output feature.

### 3.5. Loss Function

In YOLOv8, the loss function is constructed using the CIoU (Complete Intersection over Union) proposed by Zheng et al. (2020) [41], augmented with an aspect ratio penalty term derived from DIoU (Distance Intersection over Union) [42].

However, CIoU fails to account for the sample difficulty balance, and it considers the box’s aspect ratio as one of the penalty terms in the loss function. When the aspect ratios of the bounding box and the predicted box are similar but their width and height values differ significantly, this penalty term becomes largely ineffective. Equation (4) presents the formula for CIoU:

(4) $$\begin{array}{c} L_{CIoU}=1-IoU+\frac{\rho^{2}\left(b,b^{gt}\right)}{c^{2}}+\alpha \nu \\ R_{CIoU}=\frac{\rho^{2}\left(b,b^{gt}\right)}{c^{2}}+\alpha \nu \\ \alpha =\frac{\nu }{(1-IoU)+\nu }\\ \nu =\frac{4}{\pi^{2}}{\left(arctan\frac{w^{gt}}{h^{gt}}-arctan\frac{w}{h}\right)}^{2} \end{array}$$

where $L_{CIoU}$ stands for the loss function for Complete IoU (CIoU), with $R_{CIoU}$ representing the penalty term added on top of the IoU loss function. The variables *w* and *h* denote the width and height of the prediction box, respectively. The parameter ρ signifies the Euclidean distance between the computed center points. Similarly, $w^{gt}$ and $h^{gt}$ represent the width and height of the ground truth box, while *b* and $b^{gt}$ indicate the center points of the ground truth box and the prediction box. *c* represents the diagonal length of the smallest enclosing box covering the two boxes. The Intersection over Union IoU indicates the overlap between the predicted box and the ground truth box. Additionally, α acts as a balancing term that adjusts the position of the bounding box’s center point relative to its size, while ν represents the cosine of the bounding box’s aspect ratio.

To address the challenges associated with CIoU, WIoU was integrated into the network. WIoU assesses the anchor frame’s quality through the implementation of a dynamic non-monotonic mechanism and the adoption of a more rational dynamic gradient gain allocation technique. This approach diminishes the competitive advantage of high-quality anchor frames for small targets, thereby mitigating the adverse effects of low-quality anchor frames on gradient calculations. Consequently, the loss function can allocate more focus to the standard quality of small target anchor frames, thereby enhancing the overall performance of the detector. The formula for WIoU v1 is presented in Equation (5):

(5) $$\begin{array}{c} L_{WIoUv1}=R_{WIoU}\times L_{IoU}\\ R_{WIoU}=exp\left(\frac{{\left(x-x_{gt}\right)}^{2}+{\left(y-y_{gt}\right)}^{2}}{{\left(W_{g}^{2}+H_{g}^{2}\right)}^{2}}\right)\\ L_{IoU}=1-IoU \end{array}$$

In Formula (5), *x*, *y*, $x_{gt}$, and $y_{gt}$ denote the center point coordinates of the prediction box and the ground truth box. $W_{g}^{2}$ and $H_{g}^{2}$ denote the squares of the width and height of the minimum frame that encloses both the predicted box and the ground truth box. Moreover, the Intersection over Union (IoU∈[0,1]) is utilized to quantify the extent of overlap between the anchor box and the target box in object detection tasks. $L_{WIoUv1}$ represents the WIoU loss function, while $R_{WIoU}$ represents the penalty term added on top of the $L_{IoU}$ loss function. The term $R_{WIoU}$ significantly amplifies $L_{IoU}$ for anchor boxes of standard quality.

In WIoU v3, a hyperparameter β, acting as an outlier to characterize the anchor frame mass, is introduced based on the v1 iteration. This β parameter plays a pivotal role in formulating a non-monotonic focusing coefficient γ, which effectively lessens the influence of low-quality anchor frames while amplifying the significance of high-quality anchor frames. Both σ and β are adjustable parameters that can be fine-tuned to cater to various model requirements.

(6) $$\begin{array}{c} \gamma =\frac{\beta }{\sigma \alpha^{\beta -\delta }}\\ L_{WIoUv3}=\gamma L_{WIoUv1} \end{array}$$

## 4. Experiment

### 4.1. Related Indexes

In this study, evaluation metrics from the COCO dataset were utilized to examine the detection outcomes of remote sensing small targets, including Precision, Recall, mAP, and GFLOPs. These metrics are recognized as classic and widely employed indicators in the field of remote sensing.

Precision and Recall values are derived from Equation (7), where *TP* denotes the count of accurately identified small targets, *FP* signifies the count of incorrectly identified small targets, and *FN* represents the count of undetected small targets. Precision reflects the ratio of accurately detected small targets to all targets, while Recall indicates the ratio of identified small targets (inclusive of false positives) to all small targets within the training dataset.

(7) $$\begin{array}{c} P=\frac{TP}{TP+FP}\\ R=\frac{TP}{TP+FN} \end{array}$$

*mAP* is a comprehensive evaluation indicator that takes into account the accuracy of different Recall rates. emphAP can be seen as the area of the *P*-*R* curve. The curve takes the Recall rate as the horizontal axis and the accuracy rate as the vertical axis. *mAP* is the average of the area of all categories. The formula is Equation (8); the larger the area, the higher the Precision.

(8) $$\begin{array}{c} A_{AP}=\int_{0}^{1}P\left(R\right)dR\\ mAP=\frac{\sum_{i=1}^{m}AP_{i}}{m} \end{array}$$

### 4.2. Datasets

In this research, we selected VEDAI and AI-TOD, two well-known small target datasets, and partitioned them into training, testing, and validation sets in a ratio of 7:2:1. The subsequent summaries offer a concise overview of these datasets. The image dimensions were standardized to 1024 × 1024. Furthermore, we augmented the input images through a mosaic data technique. This involved randomly selecting four images from the dataset, applying transformations such as flipping, scaling, and adjusting the color space to each of these images. Subsequently, the four modified images were combined and randomly cropped to create a new composite image. This approach significantly diversifies the object detection background and enhances the model’s resilience.

**Figure 6:**
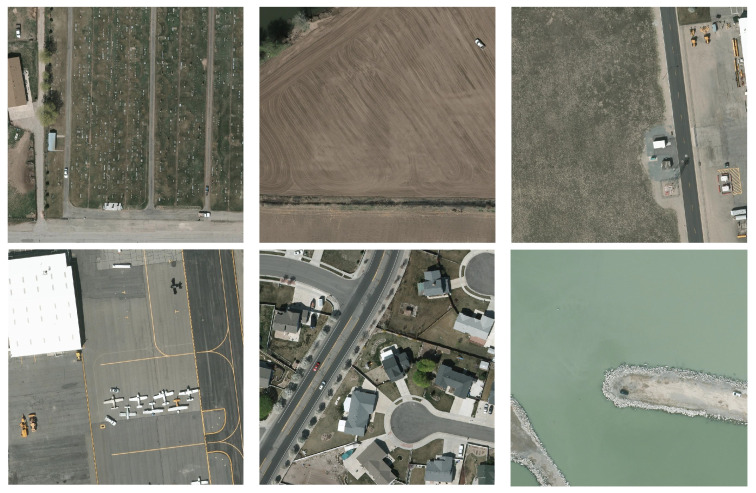
Images illustrating the different categories of the dataset [43].

**VEDAI**: VEDAI (Vehicle Detection in Aerial Imagery: A Small Target Detection Benchmark) is an aerial image dataset sourced from the Utah Automated Geographic Reference Center (AGRC). The dataset consists of cropped images with a resolution of approximately 12.5 cm by 12.5 cm per pixel. Vehicles in this dataset are characterized by their small size and diverse types. Containing over 3700 annotated objects across more than 1200 images, VEDAI exhibits variations in orientation, lighting conditions, shadows, occlusions, and other factors. The images encompass diverse backgrounds such as forests, roads, fields, and construction sites. There are nine vehicle categories present, including “plane”, “truck”, “boat”, “pick-up”, and others, as illustrated in Figure 6. Each image is available in both 1024 × 1024 and 512 × 512 dimensions, with the former size being utilized. On average, each image contains 5.5 vehicles, representing about 0.7% of the total pixel count in the image. This percentage is lower compared to many other remote sensing datasets, allowing for a comprehensive assessment of the impact of the proposed algorithm enhancements on remote sensing imagery.

**Figure 7:**
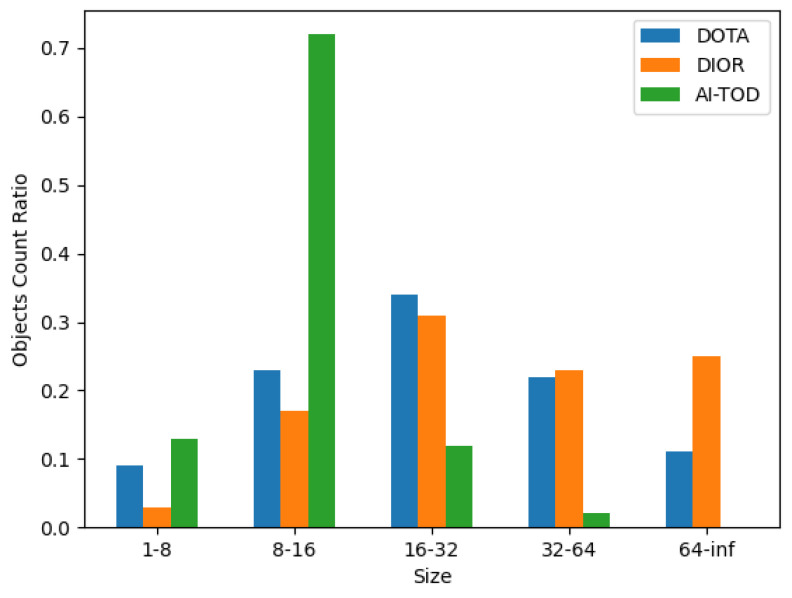
Comparison of AI-TOD with other benchmark datasets [44].

**AI-TOD**: The AI-TOD dataset, an expansive dataset enhanced in 2024 for detecting small airborne objects, is referenced [44]. This dataset is constructed upon extensive aerial image datasets, including DOTA [45], xView [46], DIOR [47], and VisDrone [48], among others. Comprising 70,621 object instances across eight categories within 28,036 aerial images, AI-TOD boasts an average target size of 12.8 pixels. Notably, as depicted in Figure 7, targets larger than 16 pixels constitute 67.8% and 79.0% in the remote sensing datasets DOTA and DIOR, respectively. Most of these datasets predominantly feature large objects, rendering them less suitable for small target detection. In AI-TOD, 85% of targets are smaller than 16 pixels, with the largest targets measuring less than 64 pixels. This dataset encompasses eight classes of object instances, including some uncommon classes like swimming pools and windmills, which are notably less prevalent compared to classes such as vehicles and boats, aligning with real-world scenarios and enhancing practical applicability.

### 4.3. Ablation Experiments

To validate the efficacy of ARSOD-YOLO, we conducted experiments on the VEDAI dataset. Building upon the results from YOLOv8n as a baseline, we systematically performed ablation experiments on various modules.

It is important to highlight that we employed the pre-training weights of YOLOv8n, as illustrated in Table 1, acquired after its training on the COCO dataset. The initial results showcase the performance of the baseline YOLOv8n, yielding a mAP50 value of 0.712 and a mAP50–95 value of 0.459. Subsequently, we iteratively added different modules. The second group showcases results after the addition of jump connections. The third group presents results post-addition of AFEM. The fourth group demonstrates results after integrating AKSFFM, showcasing a 1.4% accuracy enhancement compared to the pre-addition scenario. The fifth group showcases results from experiments combining jump connections and AKSFFM. The final set of experimental outcomes encapsulates the ARSOD-YOLO experiment.

**Figure 8:**
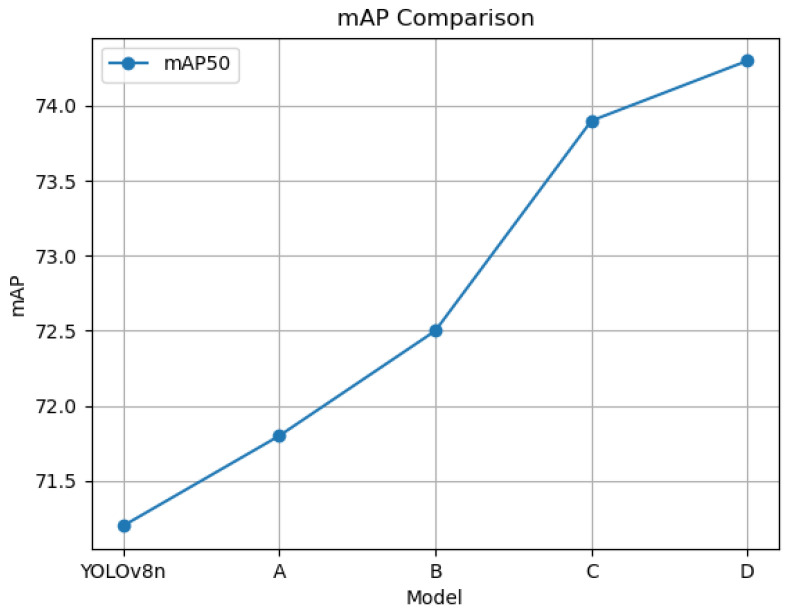
Visualization of mAP effects of different modules.

Table 1 displays the outcomes of ablation experiments conducted on the VEDAI dataset employing various modules. As the network undergoes gradual enhancements, the accuracy of target detection steadily improves. Notably, the combination of BiFPN and AKSFFM resulted in the most significant accuracy boost, showcasing a 2.3% enhancement in target detection accuracy. Upon integrating all modules, the model’s target accuracy sees a notable improvement of 3.1%. Specifically, the effects of individual module additions are as follows: The inclusion of jump connections elevates the mAP50 value by 0.6% and the mAP50–95 value by 0.2%. The integration of AFEM leads to a 0.7% improvement. Subsequent to incorporating AKSFFM, there is a 1.4% increase in the mAP50 value. These results collectively indicate a positive influence of our enhancements on the experimental outcomes. Additionally, we visualize the accuracy variations following incremental module additions using a line chart in Figure 8. In the chart, A, B, C, and D signify the magnitude of change in mAP50 after the gradual addition of BiFPN, AFEM, AKSFFM, and WIoU, illustrating a consistent enhancement in accuracy with the inclusion of each module.

### 4.4. Comparative Experiments on Loss Function

In our study, we conducted experiments on several loss functions using the VEDAI dataset in Table 2 . It is noteworthy that these experiments were carried out on network models enhanced with improvements such as BiFPN, AFEM, and AKSFFM. The table showcases the results of replacing the CIoU los4.3.2s function in YOLOv8 with SIoU, Focal_CIoU, GIoU, and WIoU, respectively. From the experimental results, it is evident that when the loss function in the network model is WIoU, the mAP50 and mAP50–95 values are maximized, with an increase of 0.8% for mAP50 and 0.6% for mAP50–95 compared to the original CIoU. Conversely, the experimental results after introducing other loss functions showed varying degrees of decline. We posit that the WIoU loss function is better suited for the characteristics and distribution of small targets in remote sensing image datasets, as it can more accurately measure the alignment between predicted and ground truth bounding boxes. Additionally, the WIoU loss function’s adaptability to different shapes through IoU calculations, unrestricted by specific shapes or sizes, enhances the generalization ability of small target detection.

### 4.5. Comparative Experiments

The table below displays the experimental results of the network model before and after enhancements on the VEDAI dataset. In Table 3, the detection outcomes for each category in the VEDAI dataset are presented. YOLOv8n represents original experimental results, while ARSOD-YOLO illustrates the detection outcomes of the improved network model. For most categories, except for car, pick-up, and boat, there was a slight decrease in the mAP50 value, whereas the mAP50 values for other categories exhibited significant improvements. Notably, the tractor category saw a substantial increase, with a remarkable 10.6% rise in mAP50. On average, there was a 3.1% increase in mAP50 across all categories. Precision also witnessed a significant boost, escalating by 5.7%. These experimental outcomes unequivocally demonstrate the substantial enhancement brought about by the proposed algorithm on the VEDAI dataset, effectively elevating the detection accuracy of small targets in remote sensing applications.

**Figure 9:**
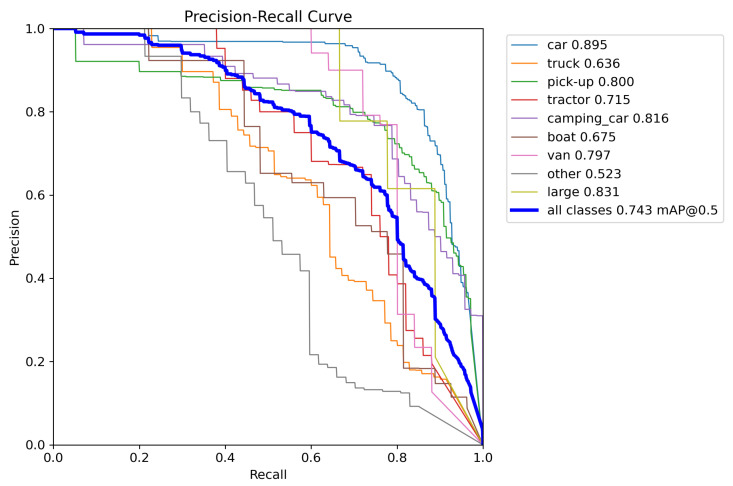
PR curves for categories in the VEDAI dataset.

Figure 9 displays the PR curves for each category in the VEDAI dataset, where P represents Precision and R represents Recall. It is evident that most categories exhibit high detection accuracies, with categories such as “pick-up”, “large”, and “camping_car” achieving over 80%.

**Figure 10:**
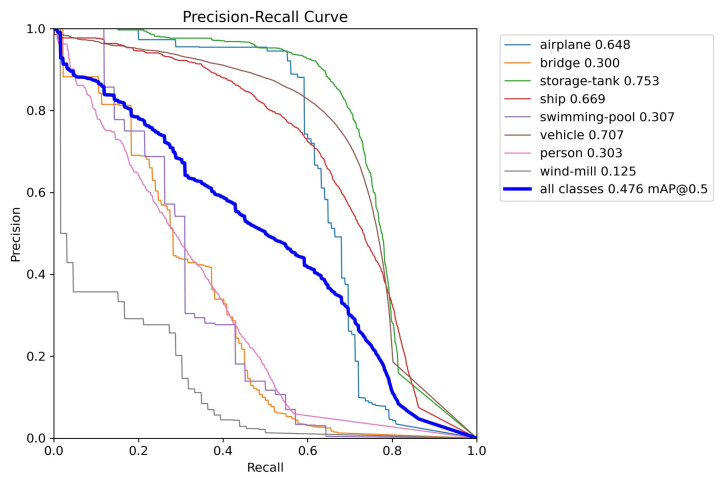
PR curves for categories in the AI-TOD dataset.

The experimental results and PR curves for the AI-TOD dataset are depicted in Table 4 and Figure 10. In the AI-TOD dataset, the enhanced model demonstrated a 6.1% increase compared to the pre-improved mAP50. With the exception of the bridge category, vehicles saw a slight decline, while other categories showed more notable improvements. Notably, the airplane category experienced a 5.9% enhancement in mAP50, and the swimming pool category saw a substantial improvement of nearly 30%. The remarkable enhancement in the swimming pool category could be attributed to its relatively small sample size; there were only 42 swimming pool targets within the test dataset. Conversely, the test set contained a considerable number of vehicle targets, reaching 61,107, with a corresponding mAP50 increase of 3.6%.

The PR curve of our proposed ARSOD-YOLO in the AI-TOD dataset is illustrated in Figure 10. The detection accuracy within the AI-TOD dataset is typically low, with an average mAP50 across all categories amounting to 0.476. The windmill category exhibited the lowest mAP50 at just 0.125, while the storage-tank category displayed the highest at 0.753.

To validate the performance of ARSOD-YOLO, we conducted a comparison with various mainstream detection models using the VEDAI dataset. As shown in Table 5, the mAP50 and mAP50–95 metrics for YOLOv8 are 71.2% and 46.2%, respectively, with a GFLOPs of 8.1. Following enhancements, the improved model exhibited increased GFLOPs at 25.1, with mAP50 and mAP50–95 values reaching 74.3% and 46.9%, respectively, marking a 3.4% mAP50 improvement. In the first group of experiments, the YOLOv3-t algorithm achieved detection accuracies of 55.3% for mAP50 and 30% for mAP50–95. Compared to the YOLOv5n algorithm in the second experimental group, although the Precision value slightly increased, other accuracy metrics decreased, alongside reduced corresponding GFLOPs. The upgraded YOLOv9t model demonstrated inferior detection accuracy compared to YOLOv8n, with both mAP values showing lower and higher GFLOPs than YOLOv8n and ARSOD-YOLO. Furthermore, YOLOv10 achieved mAP50 and mAP50–95 scores of 72.6% and 46.5%, respectively, showcasing improved accuracy compared to YOLOv8n but still falling short by 1.7% and 0.4% in comparison to our approach. Algorithms based on Transformers, such as RT-DETR and TPH-YOLO, displayed lower accuracy than our proposed method, with significantly higher GFLOPs. Furthermore, single-stage SSD and two-stage Fast-RCNN algorithms also exhibited lower accuracy levels compared to our proposed algorithm.

To assess the detection accuracy of ARSOD-YOLO on the AI-TOD dataset, a comparative experiment was conducted. Table 6 presents the detection accuracy of ARSOD-YOLO alongside several other algorithm models, along with the associated GFLOPs requirements. The results for the YOLO series are detailed in the table. In terms of detection accuracy, ARSOD-YOLO consistently outperforms other algorithms, with YOLOv10n following closely, albeit excelling in being lightweight. The mAP value of the Transformer-based algorithm is significantly lower than that of our designed algorithm, and the parameter count is notably higher compared to our proposed approach. When compared to the upgraded versions of YOLOv8n, namely, YOLOv9t and YOLOv10n, our algorithm also demonstrates superior detection accuracy.

### 4.6. Visual Experiment on VEDAI Dataset

In our study, we conducted a comparative analysis between ARSOD-YOLO and YOLOv3, YOLOv5, and YOLOv10. Four images depicting diverse scenes and targets from the VEDAI dataset were selected for evaluation. The detection outcomes were visually represented using distinct color-coded bounding boxes, with category labels and confidence scores annotated on each bounding box.

**Figure 11:**
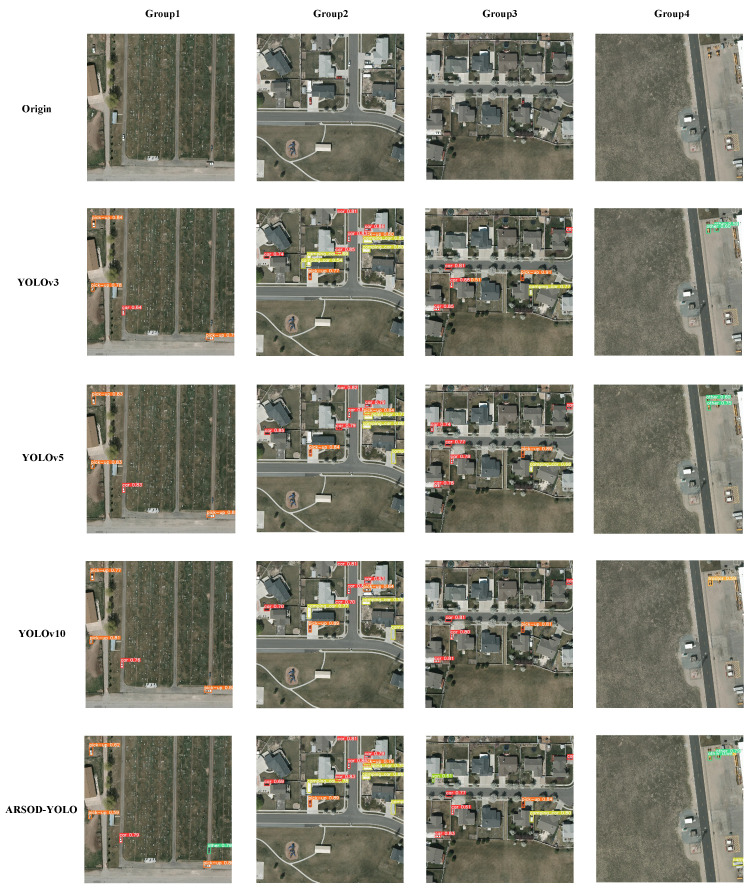
Visual comparison of object detection models: ARSOD-YOLO vs. YOLOv3, YOLOv5, and YOLOv10 on VEDAI dataset images.

As illustrated in Figure 11, our findings revealed notable disparities among the models.

For Group 1, among the models evaluated, only ARSOD-YOLO successfully identified a distinct class of objects highlighted in green, a task which eluded the other models. This underscores ARSOD-YOLO’s proficiency in detecting seemingly inconspicuous targets, thereby exhibiting superior detection accuracy. For group 2, YOLOv3, YOLOv5, and YOLOv10 failed to detect the camping_car across different positions, while ARSOD-YOLO accurately detected it. This underscores ARSOD-YOLO’s efficacy in recognizing challenging targets where other models faltered. For group 3, YOLOv10 missed detecting a van, while YOLOv5 misclassified it as a car. In contrast, both YOLOv3 and ARSOD-YOLO correctly identified the van, showcasing their robust detection capabilities. For group 4, ARSOD-YOLO demonstrated comprehensive detection by successfully identifying all targets in the images, whereas the other algorithms exhibited varying degrees of missed detections and misclassifications. These results underscore the superior detection performance of ARSOD-YOLO across diverse scenarios, outperforming YOLOv3, YOLOv5, and YOLOv10 in challenging detection tasks.

## 5. Discussion

The experimental findings on the VEDAI and AI-TOD datasets underscore the superior performance of ARSOD-YOLO in contrast to existing object detection algorithms. Nevertheless, our experiments have brought to light certain limitations, revealing that not all classes demonstrate improved accuracy when specific modules are combined as opposed to when utilized individually. Future research endeavors will concentrate on fine-tuning hyperparameters, refining network structures, and addressing these identified issues. Furthermore, while our study leveraged publicly available datasets, upcoming efforts will strive to develop proprietary datasets or enhance existing ones to facilitate data augmentation. This may involve simulating images under diverse weather conditions and lighting scenarios to enrich the training data. Moreover, plans are in progress to further refine the model’s lightweight attributes, aiming to enhance its efficiency and applicability in real-world remote sensing applications.

## 6. Conclusions

The detection of small targets in remote sensing images is often hampered by insufficient features, resulting in feature loss and heightened sensitivity to positional deviations during continuous convolution. In response to these challenges, we propose the ARSOD-YOLO network model, an advanced iteration building upon YOLOv8n. This model introduces significant enhancements in network architecture, modules, and loss functions. Drawing inspiration from the BiFPN concept, we integrate skip connections to retain crucial information. Introducing the innovative feature enhancement module, AFEM, allows for adaptive adjustment of feature weights and management of multiple branches, effectively addressing feature scarcity and enhancing discrimination between remote sensing backgrounds and targets. Furthermore, we incorporate the AKSFFM module to leverage attention weights at various scales, facilitating the learning of feature dependencies and enabling improved fusion of small target features. This approach helps mitigate information loss challenges encountered during convolution in remote sensing scenarios. To enhance the precision of small target localization, we utilize WiSe-IoU as a loss function. Our proposed ARSOD-YOLO enhances the YOLOv8 network to tackle the challenge of low accuracy in detecting small targets in remote sensing. Experimental results on the VEDAI dataset and AI-TOD dataset showed that ARSOD-YOLO achieves a favorable balance between precision and speed. The experimental results on the VEDAI and AI-TOD datasets, as well as comparisons with several popular object detection algorithms, indicate that ARSOD-YOLO achieves a good balance between accuracy and speed.

## Figures and Tables

**Table 1:** Ablation experiments on VEDAI dataset.

Dataset	BiFPN	AFEM	AKSFFM	WIoU	mAP50 (%)	mAP50–95 (%)
VEDAI	×	×	×	×	0.712	0.459
	✓	×	×	×	0.718	0.461
	✓	✓	×	×	0.725	0.472
	✓	✓	✓	×	0.739	0.466
	×	×	×	✓	0.729	0.469
	✓	×	✓	×	0.735	0.463
	✓	✓	✓	✓	0.743	0.469

**Table 2:** Comparative experiments on loss function.

Method	mAp50	mAp50–95
CIoU	0.735	0.463
SIoU	0.704	0.449
Focal_CIoU	0.734	0.455
GIoU	0.729	0.46
WIoU	0.743	0.469

**Table 3:** Comparison of target detection results before and after improvement on the VEDAI dataset.

Model	Categories	P (%)	R (%)	mAP50 (%)
YOLOv8n	car	0.787	0.87	0.922
	truck	0.537	0.571	0.616
	pick-up	0.692	0.836	0.853
	tractor	0.715	0.6	0.609
	camping_car	0.726	0.784	0.786
	boat	0.499	0.667	0.706
	van	0.636	0.699	0.759
	other	0.448	0.383	0.399
	large	1	0.617	0.756
	all	0.671	0.67	0.712
ARSOD-YOLO	car	0.82	0.84	0.895
	truck	0.638	0.543	0.636
	pick-up	0.686	0.834	0.8
	tractor	0.74	0.568	0.715
	camping_car	0.694	0.789	0.816
	boat	0.623	0.63	0.675
	van	0.819	0.72	0.797
	other	0.611	0.468	0.523
	large	0.923	0.667	0.831
	all	0.728	0.673	0.743

**Table 4:** Comparison of target detection results before and after improvement on the AI-TOD dataset.

Model	Categories	P (%)	R (%)	mAP50 (%)
YOLOv8n	vedaiself	0.434	0.616	0.589
	bridge	0.562	0.334	0.331
	storage-tank	0.843	0.652	0.739
	ship	0.603	0.64	0.629
	swimming-pool	1	0.0335	0.0604
	vehicle	0.672	0.681	0.671
	person	0.671	0.282	0.26
	windmill	0.397	0.0152	0.0591
	all	0.616	0.407	0.417
ARSOD-YOLO	airplane	0.714	0.6	0.648
	bridge	0.518	0.289	0.307
	storage-tank	0.849	0.671	0.748
	ship	0.731	0.596	0.668
	swimming-pool	0.439	0.31	0.307
	vehicle	0.746	0.682	0.707
	person	0.535	0.267	0.307
	windmill	0.331	0.182	0.136
	all	0.608	0.45	0.478

**Table 5:** Comparative experiment on VEDAI dataset.

Method	P (%)	R (%)	mAP50 (%)	mAP50–95 (%)	FLOPs (G)
YOLOv3t	0.51	0.564	0.553	0.3	13
YOLOv5n	0.602	0.511	0.517	0.302	5.9
YOLOv9t	0.817	0.591	0.654	0.429	11.1
YOLOv8n	0.671	0.67	0.712	0.462	8.1
YOLOv10n	0.729	0.658	0.726	0.465	8.3
RT-DETR	0.455	0.436	0.455	0.436	103.56
Fast-RCNN	-	-	0.459	-	196.2
SSD	-	-	0.451	-	-
TPH-YOLO	-	-	0.584	0.338	270.9
Ours	0.728	0.673	0.743	0.469	25.1

**Table 6:** Comparative experiment on AI-TOD dataset.

Method	P (%)	R (%)	mAP50 (%)	mAP50–95 (%)	FLOPs (G)
YOLOv3t	0.475	0.298	0.309	0.131	13.1
YOLOv5n	0.698	0.311	0.311	0.342	6.1
YOLOv8n	0.616	0.407	0.417	0.18	8.2
YOLOv9t	0.592	0.277	0.384	0.299	12
YOLOv10n	0.538	0.203	0.455	0.203	8.2
RT-DETR	0.614	0.229	0.191	0.121	108.1
HANet	-	-	0.529	0.210	-
Ours	0.608	0.45	0.478	0.209	25.3

## Data Availability

The original contributions presented in the study are included in the article; further inquiries can be directed to the corresponding author.
